# Predicting Clinical Scores from Magnetic Resonance Scans in Alzheimer's Disease

**DOI:** 10.1016/j.neuroimage.2010.03.051

**Published:** 2010-03-25

**Authors:** Cynthia M. Stonnington, Carlton Chu, Stefan Klöppel, Clifford R Jack, John Ashburner, Richard S.J. Frackowiak, the Alzheimer Disease Neuroimaging Initiative

**Affiliations:** 1 Department of Psychiatry and Psychology, Mayo Clinic, Scottsdale, AZ, USA; 2 Wellcome Trust Centre for Neuroimaging, Institute of Neurology, University College London, London, United Kingdom; 3 Section on Functional Imaging Methods, Laboratory of Brain and Cognition, NIMH, NIH; 4 Department of Psychiatry and Psychotherapy, section of Gerontopsychiatry and Neuropsychology and Freiburg Brain Imaging, University Clinic Freiburg, Freiburg, Germany; 5 Department of Radiology, Mayo Clinic, Rochester, MN, USA; 6 Service de Neurologie, Centre Hospitalier Universitaire Vaudois, 46 rue du Bugnon, 1011-Lausanne, CH; 7 Laboratory of Neuroimaging, IRCCS Santa Lucia, Roma, Italy

**Keywords:** Alzheimer's disease, multivariate, machine learning, relevance vector regression, MMSE, DRS, AVLT, ADAS-Cog

## Abstract

Machine learning and pattern recognition methods have been used to diagnose Alzheimer's disease (AD) and Mild Cognitive Impairment (MCI) from individual MRI scans. Another application of such methods is to predict clinical scores from individual scans. Using relevance vector regression (RVR), we predicted individuals' performances on established tests from their MRI T1 weighted image in two independent datasets. From Mayo Clinic, 73 probable AD patients and 91 cognitively normal (CN) controls completed the Mini-Mental State Examination (MMSE), Dementia Rating Scale (DRS), and Auditory Verbal Learning Test (AVLT) within 3 months of their scan. Baseline MRI's from the Alzheimer's disease Neuroimaging Initiative (ADNI) comprised the other dataset; 113 AD, 351 MCI, and 122 CN subjects completed the MMSE and Alzheimer's Disease Assessment Scale—Cognitive subtest (ADAS-cog) and 39 AD, 92 MCI, and 32 CN ADNI subjects completed MMSE, ADAS-cog, and AVLT. Predicted and actual clinical scores were highly correlated for the MMSE, DRS, and ADAS-cog tests (*P*<.0001). Training with one dataset and testing with another demonstrated stability between datasets. DRS, MMSE, and ADAS-Cog correlated better than AVLT with whole brain grey matter changes associated with AD. This result underscores their utility for screening and tracking disease. RVR offers a novel way to measure interactions between structural changes and neuropsychological tests beyond that of univariate methods. In clinical practice, we envision using RVR to aid in diagnosis and predict clinical outcome.

## Introduction

With no single marker yet available, combining different relevant data is one proposed way to increase diagnostic power for Alzheimer's disease (AD). In particular, the combination of neuropsychological and neuroimaging data makes sense, since preclinical AD has been associated with both cognitive and imaging changes (Caselli et al., 2009; Caselli et al., 2007; Reiman et al., 1996; Reiman et al., 2009; Twamley et al., 2006). After a diagnosis of mild cognitive impairment (MCI) or AD, the combination of cognitive tests and imaging can be used for both tracking progression of illness and treatment response. For all of these purposes, the ideal neuropsychological tests must closely reflect the atrophy patterns of the disease. In this paper we focus on a novel method to correlate an individual's test score with structural changes.

The relationship between commonly used cognitive measures and structural changes with MRI has been previously examined using voxel based morphometry (VBM) (Baxter et al., 2006; Jack et al., 2008b) and region of interest studies (Apostolova et al., 2006; Duchesne et al., 2009; Fama et al., 1997; Ferrarini et al., 2008; Jack et al., 2004). Duchesne and colleagues (Duchesne et al., 2009) demonstrated a relationship between baseline MRI features and decline in Folstein Mini-Mental State Exam (MMSE) after one year using linear regression modeling within a volume of interest in subjects with MCI. VBM separately compares the volume of tissue around each point or voxel of the whole brain, has the advantage of not being biased to one particular region or structure (Ashburner and Friston, 2000), and is very useful for examining group differences. Though VBM can be applied to single subject data through comparisons of individual scans with those in a normal control group (Chetelat et al., 2008), the statistical assumptions underlying such a procedure are not without problems so that single subject analysis with VBM is limited in its scope in terms of translation to the clinic for individual patients.

Pattern recognition and machine learning methods (Bishop, 2006; Tipping, 2001; Vapnik, 1998) offer another way to analyze MRI scans at the single-subject level. Multivariate pattern recognition techniques take into account specific inter-regional dependencies characteristic of different distributed pathologies, using such information to help separate the data (Lao et al., 2004). So far, most applications of pattern recognition to imaging data have been for binary classification, where the objective has been to predict which class a subject belongs to. In particular, support vector machines (SVM) have been used to classify AD (Davatzikos et al., 2008a; Davatzikos et al., 2008b; Gerardin et al., 2009; Kloppel et al., 2008; Vemuri et al., 2008) and progression to AD from MCI (Davatzikos et al., 2009; Plant et al. 2010). In this paper, the objective is to predict a continuous measure from the MR scans, i.e., a clinical score of dementia severity, after modeling the probability of the clinical scores given the data. Evidence suggests that Relevance Vector Regression (RVR; Tipping, 2001) generally performs well for such problems. The Pittsburgh brain activity interpretation competition (http://www.lrdc.pitt.edu/ebc/PBAIC.html) provided objective comparisons among a number of regression methods, and RVR was found to perform well for fMRI data. The winning team (C.C. and J.A. from the current study were on this team; http://www.lrdc.pitt.edu/ebc/2007/2007.html) as well as one of the second place teams both used RVR. Recently, Franke and colleagues (Franke et al., 2010) reported using RVR and support vector regression (SVR) for prediction of age of healthy subjects from MR structural scans and found that RVR performed very well and better than SVR for age predictions.

We applied RVR to the prediction of clinical ratings based on the MR structural images from two independent data bases and four commonly used cognitive measures: the MMSE (Folstein et al., 1975), which examines orientation to time and place, immediate and delayed recall of three words, attention and calculation, language and visuo-constructional functions; the Mattis Dementia Rating Scale (DRS) (Mattis, 1988), which tests the areas of attention, initiation/perseveration, construction, memory, and conceptualization; the Alzheimer's Disease Assessment Scale—Cognitive subtest (ADAS-cog), a global measure encompassing the core symptoms of AD (Rosen et al., 1984); and Rey's Auditory Verbal Learning Test (AVLT) (Rey, 1964), a measure of verbal memory. We reasoned that the cognitive test with the greatest predictive accuracy overall would closely reflect whole brain structural changes associated with AD, whereas tests measuring a single cognitive domain, e.g., AVLT, would perform less well.

## Methods

We applied RVR to three sets of subjects. “Set 1” were patients with probable AD and cognitively normal (CN) controls from the Mayo Rochester Alzheimer's Disease Research Center (ADRC) and Mayo Alzheimer's Disease Patient Registry (ADPR) (Petersen et al., 1990) who had all three MMSE, DRS, and AVLT scores recorded within three months of their MRI scan. Of 200 subjects, 10 scans were excluded for artifacts and abnormalities. From the remaining 190 subjects, 164 had all three MMSE, DRS, and AVLT scores recorded within three months of the scan. “Set 2” were probable AD, MCI, and CN subjects downloaded from the freely available Alzheimer's Disease Neuroimaging Initiative (ADNI) database (www.loni.ucla.edu/ADNI) who had both the MMSE and ADAS-Cog scores recorded within three months of their MRI scan. “Set 3” were probable AD, MCI, and CN subjects from the ADNI database who had all three MMSE, ADAS-Cog, and AVLT scores recorded within three months of their MRI scan. Of 610 subjects with baseline MRI scans in the ADNI database, 12 were excluded for artifacts and abnormalities. From the remaining 598 subjects, 586 had both the MMSE and ADAS-Cog scores recorded (“Set 2”), and 163 had the MMSE, ADAS-Cog and AVLT recorded (“Set 3”). Details of the group are outlined in Table 1. Because the aim was to pair the score with the image, rather than distinguish between groups, it was not necessary to exclude control subjects with MMSE<27 or for patients and controls to be exactly age and education-matched. Subjects were excluded from analysis if their scan revealed gross structural abnormalities other than atrophy. In order to be included in this study, subjects had to have clinical scores recorded within three months of their MRI scan.

### Set 1

Participants were 73 patients with probable AD, ranging from mild to severe (MMSE from 10 to 30, mean 22.3) and 91 CN controls from the Mayo Rochester ADRC and Mayo ADPR (Petersen et al., 1990). The diagnosis of probable AD was made according to the DSM-III-R(1987) and NINCDS-ADRDA criteria for AD (McKhann et al., 1984). Cognitively normal (CN) controls were deemed to be normal by clinical history and examination, including eight controls with MMSE scores less than 27.

MR scans were collected over a period of about 10 years with a total of 14 different scanners. Several software updates occurred at different times for different scanners. However, a closely followed quality control program insured uniformity over time. All scanners were monitored with daily phantom quality checks, which calibrated the gradients to within +/- 1 mm over a 240 mm rectangular field of view, and with signal to noise and radio frequency transmit gain. All scans were done on the same platform, General Electric Signa 1.5T scanners (T1-weighted image parameters: TR= 17.7 to 27 ms, TE= 6 to 10 ms, flip angle 25° or 45°, voxel size 0.86 mm × 0.86 mm × 1.6 mm, matrix dimensions 256 × 192). The major hardware elements (body resonance module gradient coil and birdcage head transmit-receive volume coil) were unchanged throughout time and across all scanners. Separate VBM analyses, described elsewhere (Stonnington et al., 2008) showed no significant interaction of scanner or upgrade with the effect of disease.

Patients and controls were matched equally for sex, but controls were slightly younger than patients (mean age 74.7 vs. 77.3; *P* = .04) and slightly more educated (mean years 14.6 vs. 13.5; *P*=.01).

### Set 2

113 AD, 351 MCI, and 122 CN subjects from the ADNI database (www.loni.ucla.edu/ADNI) had both MMSE and ADAS-Cog scores recorded. The primary goal of ADNI has been to test whether serial MRI, PET, other biological markers and clinical and neuropsychological assessment can be combined to measure the progression of MCI to early AD.

The general inclusion criteria were those of ADNI (http://clinicaltrials.gov/show/NCT00106899). According to ADNI clinical procedures, a diagnosis of AD was made if the subject had a MMSE score between 20 and 26, a Clinical Dementia Rating scale (Morris, 1993) score of 0.5 or 1, and met NINCDS/ADRDA (McKhann et al., 1984) criteria for probable Alzheimer's disease. Individuals were classified as single-domain amnestic MCI if they satisfied the following criteria: (i) score on the MMSE between 24 and 30; (ii) Clinical Dementia Rating scale = 0.5; (iii) reported memory complaint; (iv) objective memory loss measured by education-adjusted scores on Wechsler Memory Scale Logical Memory (Wechsler, 1987); (v) absence of significant levels of impairment in other cognitive domains; (vi) preserved activities of daily life; and (vii) absence of dementia. Healthy controls had an MMSE score between 24 and 30 and a Clinical Dementia Rating scale score of 0. Whatever the inclusion group, subjects had a Geriatric Depression Scale score of less than 6.

Baseline MRI's were downloaded from the ADNI dataset. ADNI is a multicenter project that combines data from 55 participating sites and includes MRI data from 3 different vendors (GE Healthcare, Philips Medical Systems, or Siemens Medical Solutions/http://www.loni.ucla.edu/ADNI/Data/ADNI_Data.shtml) (Jack et al., 2008a). The detailed protocol for each scanner is publicly available (http://www.loni.ucla.edu/ADNI/Research/Cores/). A sophisticated system for quality control including phantom scanning is in place (Gunter et al., 2009; Mortamet et al., 2009). The target voxel size is approximately 1 mm^3^, with a maximum of 1.5 mm in any one direction. All of the MRI data in this study were acquired on a 1.5 T scanner.

CN, MCI, and AD were matched for age, but AD patients were less educated than MCI and CN subjects (mean years of education AD=14.6, MCI=15.8, CN=15.9, *P*=.0006) and the MCI subjects had more men than the AD subjects (AD=52%, MCI=65%, CN=56%, *P*=.03). Among the MCI subjects, 220 did not convert to AD during the follow-up period, and 131 converted to AD (mean 517.5 days, range 172 to 1111 days). Spearman's correlation coefficient was used to compare the correlations of predicted and actual scores with days until AD conversion and to compare the correlations of years of education with predication accuracy (predicted score minus the actual score).

### Set 3

Only 39 AD, 92 MCI, and 32 CN subjects from the ADNI database had all three MMSE, ADAS-Cog and AVLT scores recorded at baseline. Age and sex was not significantly different in the AD, MCI and CN groups, but the AD group was slightly less educated (mean years of education AD=14.2, MCI=15.8, CN=15.8, *P*=.03).

### Cross-validation of Set 1 and 2

In order to test the stability of the method with independent data sets, we created a training set with Set 1 and tested with Set 2, and *vice versa*. We also examined the importance of having a wide range of disease severity represented in the training sets by training and testing with and without the MCI group.

### Image Processing (Figure 1)

Images were visually inspected for artifacts or structural abnormalities unrelated to AD. Images were firstly segmented into white and grey matter (GM) and cerebrospinal fluid using the “unified segmentation” (Ashburner and Friston, 2005) approach in SPM5 for Set 1 and SPM8 for Set 2 (Wellcome Trust Centre for Neuroimaging, Institute of Neurology, UCL, London UK – http://www.fil.ion.ucl.ac.uk/spm). Then, GM segments were further normalized to a population template generated from the complete image set using a diffeomorphic registration algorithm (DARTEL; Ashburner, 2007). This non-linear warping technique minimizes structural variation between subjects and has been shown to be more accurate than the standard approach to normalization implemented in SPM (Bergouignan et al., 2009; Klein et al., 2009). A separate Jacobian transformation step (often referred to as ‘modulation’) that multiplies the partitioned images by the relative voxel volumes, *i.e.*, the Jacobian determinants of the deformation field (Ashburner and Friston, 2000), was used to ensure that the overall volume of each tissue class remained constant after normalization. As in our previous work using SVM and DARTEL normalisation (Kloppel et al., 2008), no spatial smoothing was performed for our primary analyses because of the greater accuracy of the DARTEL method. However, a post-hoc analysis that included spatial smoothing with a 6 mm Gaussian kernel was performed to confirm this supposition. The voxels in the pre-processed data served as features for subsequent pattern recognition.

### Relevance Vector Regression

Relevance Vector Regression (RVR) is a sparse kernel method formulated in a Bayesian framework (Tipping, 2001; Bishop, 2006). Unlike typical kernel algorithms such as support vector machines (SVM), RVR treats the kernel as a set of linear basis functions in order to obtain the form of equation. *φ*:x_*_ ∈ ℜ*^D^* → *φ*(x_*_) = (*k*(x_*_,**x**_1_),….,*k*(x_*_,**x***_N_*)) ∈ ℜ*^N^*, where k(x1,x2) is the kernel function, *D* is the dimensionality of a feature set and *N* is the number of samples. In this particular work, it is defined as the dot product of two input vectors. The ‘kernel matrix’ **K** is generated from the pre-processed image data (*i.e.*, spatially normalized and modulated GM segments). The set of features from each individual subject (in our case, these features were voxel values) can be considered as a point located in a high dimensional space, where the number of features determines the number of dimensions. For linear regression models, the pairwise similarity measures are computed from the dot-product of each image, with every other image. Computing a dot-product of an image pair simply involves multiplying the voxel values of one image by the values of the corresponding voxels of the other, and adding up the result.

Strictly speaking, RVR is not a kernel algorithm because its input is not required to be a kernel satisfying Mercer's condition. In other words, the input matrix need not be symmetric and positive definite matrix. It is also possible to take only a few “representative samples”, and use similarity measures, *i.e.* kernel values, or dissimilarity measures of these samples as basis functions. The general RVM takes a full kernel matrix as input, and appends a column of ones to model the offset. In this work, we apply this standard formulation. We will denote the *N* by *N*+1 basis functions by **Φ** = [**l, K**], where **l** is an *N* element column vector of ones.

The likelihood function of the data set can be modeled by a Gaussian distribution, *p*(**t** | **w,*σ***^2^) = *N*(**t** | **Φw, *σ***^2^**I**), where **t** is the target variable. Each of the weights, **w**, are modeled to have a zero mean Gaussian prior with independent variance $\alpha_{i}^{-1}$, so the weight prior is $p(\text{w}|\text{α})=\overset{N}{\underset{i=0}{\text{∏}}}N({\text{w}}_{i}|0,\alpha_{i}^{-1})$. Combining the prior and likelihood functions, yields the posterior distribution over the weights, *p*(**w** | **t,α,σ**^2^) = *N*(**w** | **μ,Σ**), where **Σ** = (*σ*^−2^**Φ^T^Φ** + **A**)^−1^ is the posterior covariance and **A** = *diag*(*α*_0_,*α*_1_,…, *α_N_*) is the diagonal matrix with the precision, or the inverse of the variance, for each weight. **μ** = *σ*^−2^**ΣΦ^T^t** is the maximum posterior weight. In the Bayesian framework, finding an optimum solution involves maximising the marginal likelihood (type-II maximum likelihood) with respect to the hyper-parameters **α** and a noise variance *σ*^2^. Because both the likelihood and the prior are modeled by Gaussian distributions, it is analytically feasible to derive the marginal likelihood function by integrating over the parameters (weights). The marginal likelihood is also a Gaussian *p*(**t** | **∫,σ**^2^) = ∫ *p*(**t** | **w, σ**^2^)*p*(**w** | **α**)*d***w** = *N*(**t** | 0,**C**), where **C** = *σ*^2^**I** +**ΦA**^−1^**Φ^T^** is the covariance of the marginal likelihood. The objective of the optimisation is to find the hyper-parameters, **A**,**σ**^2^, which maximise the “evidence” of the data. When maximising the marginal likelihood, some of the *α* will grow very large, implying a small prior variance. Because the prior is zero mean, a parameter with an extremely small variance will have its posterior probability sharply peaked at zero. This property allows irrelevant columns of basis functions to be pruned out, and is known as automatic relevance determination (ARD) (MacKay, 1995). The parameters with non-zero weights are called “relevance vectors”, which are analogous to “support vectors.”

**Fig. 2:**
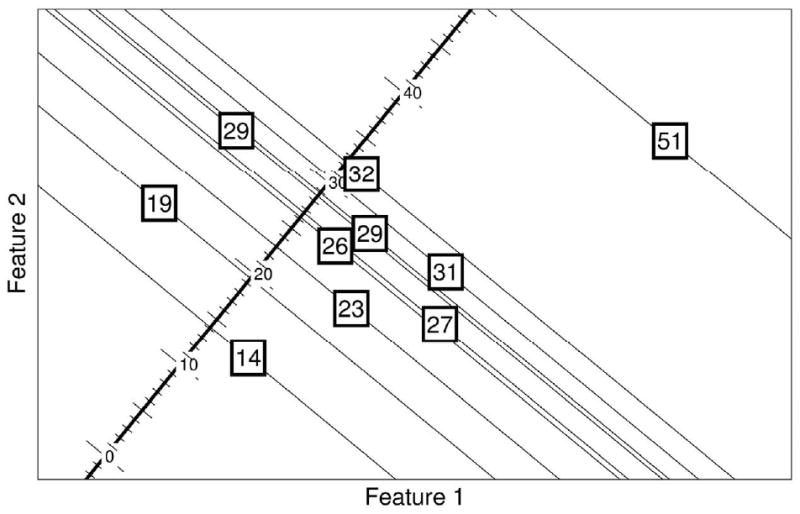
Illustration of Relevance Vector Regression with hypothetical 2D training data. The numbers in the squares are the training targets, i.e., actual scores, and the coordinates show the value of 2 different voxel intensities (feature 1 and feature 2). The goal is to find the features most predictive of the clinical score. All subjects are projected into a 1D line in such a way that minimizes the differences between the actual scores and the scores after projection, i.e., predicted score. For example, box 31 is projected into a value less than 31, which is the error that needs to be minimized in the learning algorithm.

The training step enables an RVR to use basis functions **Φ** to learn the relationship between images and corresponding clinical scores. Figure 2 is a simplified illustration of linear regression. With a linear pattern recognition algorithm, it is possible to learn a weighting image, *i.e.*, the contribution of different areas of the brain in determining behavioral test scores. The predicted score of a new subject can be calculated by computing the dot-product (sum of element-wise multiplication) of the weighting image obtained from the training group and the new subject's pre-processed image (features), then adding a constant.

In order to evaluate the performance of a RVR, cross-validation was done by leaving one of the subjects out so that the remaining subjects (n-1) were used to train the RVR. The clinical rating of the subject left out was predicted from the corresponding image. This procedure was repeated for each subject and finally, a correlation was calculated between subjects' true clinical scores and the ones predicted from scan analysis by the RVR method. In addition to the leave one out method, we also cross-validated by training with one set and testing with an independent dataset. The RVR was used to test whether any of the recorded clinical scores (DRS, MMSE, ADAS-Cog and AVLT) separately and significantly correlated with structural changes in the group of AD, MCI, and CN individuals. The aim was to identify GM structures showing a monotonic relationship with clinical score. The RVR provides a prediction of the clinical score in a given test that is based on individual brain structure. The predicted and actual clinical scores were compared using Pearson's correlation coefficient, a standard technique for measuring linear relationships. The higher the correlation, the more accurate are the predictions. We also calculated the root mean square (RMS) of the errors, which are very close to the standard deviations of the errors if the mean of the errors are approximately zero. The drawback of using the RMS of the errors as the measure of prediction accuracy is that different clinical scores have different scales. In order to compare across different scores, we normalized the actual scores to have zero means and variances of one. The higher the RMS, the less accurate are the predictions.

## Results

### Set 1

For whole brain images, the correlations of predicted and actual scores were as follows: MMSE: 0.70; DRS: 0.73; AVLT: 0.60. No improvement in accuracy occurred with spatial smoothing: MMSE: 0.66; DRS: 0.72; AVLT: 0.55. The likelihood of each of these correlations occurring by chance is *P* <.0001. When restricting analysis to the 73 AD subjects only, MMSE (r=.44) and DRS (r=.54) correlations remain highly significant (*P* <.0001), but not with AVLT (r=0.16, P=0.17). The RMS of the errors were as follows: MMSE: 3.22; DRS: 10.24; AVLT: 2.85. The normalized RMS were as follows: MMSE: 0.72; DRS: 0.69 AVLT: 0.8.

### Set 2

Correlations of the predicted and actual scores for the group of 586 subjects with both MMSE and ADAS-Cog baseline scores were as follows: MMSE: 0.48, *P* <.0001; ADAS-Cog: 0.57, *P* <.0001. As in Set 1, no substantial improvement was obtained with spatial smoothing: MMSE r=0.44; ADAS-Cog r=0.58. Prediction accuracy (actual score minus predicted score) and years of education were significantly correlated for both MMSE (r=0.18, *P*<0.0001) and ADAS-Cog (r=-0.16, P <0.0001). In the subset of 351 MCI subjects, both the predicted and actual scores significantly correlated with days to conversion to AD (using 1500 days for non-converters): Actual MMSE, r=0.19, *P*=.0004; predicted MMSE, r=0.36, *P*<0.0001. Actual ADAS-cog, r=-0.37, *P*<0.0001, predicted ADAS-cog, r=-0.41, *P*<0.0001. The RMS of the errors were as follows: MMSE: 2.2; ADAS-Cog: 7.1. The normalized RMS were as follows: MMSE : 0.88; ADAS-Cog: 0.82.

### Set 1 and Set 2 Cross-validation (MMSE)

When Set 1 MMSE was used to train, and Set 2 MMSE without MCI was used to test, r=0.56, *P* <.0001. Similarly, when Set 2 without MCI was used for training and Set 1 was used for testing, r=0.60, *P* <.0001. Likewise, when training with Set 2 (including MCI) and testing with Set 1, r=0.62, *P* <.0001. However, training with Set 1 (no MCI) and testing with Set 2 (including MCI) reduced the accuracy to r=0.40.

### Set 3

For the subset of subjects with AVLT, MMSE and ADAS-Cog (n=163) all recorded, the correlations between predicted and actual scores were: MMSE 0.47 (*P* <.0001), ADAS-Cog 0.49 (*P* <.0001); AVLT percent retention 0.13 (*P* =0.10). With spatial smoothing, the correlations were: MMSE 0.51(*P* <.0001), ADAS-cog 0.48 (*P* <.0001), and AVLT r=0.17(*P* =0.03).

**Fig. 3:**
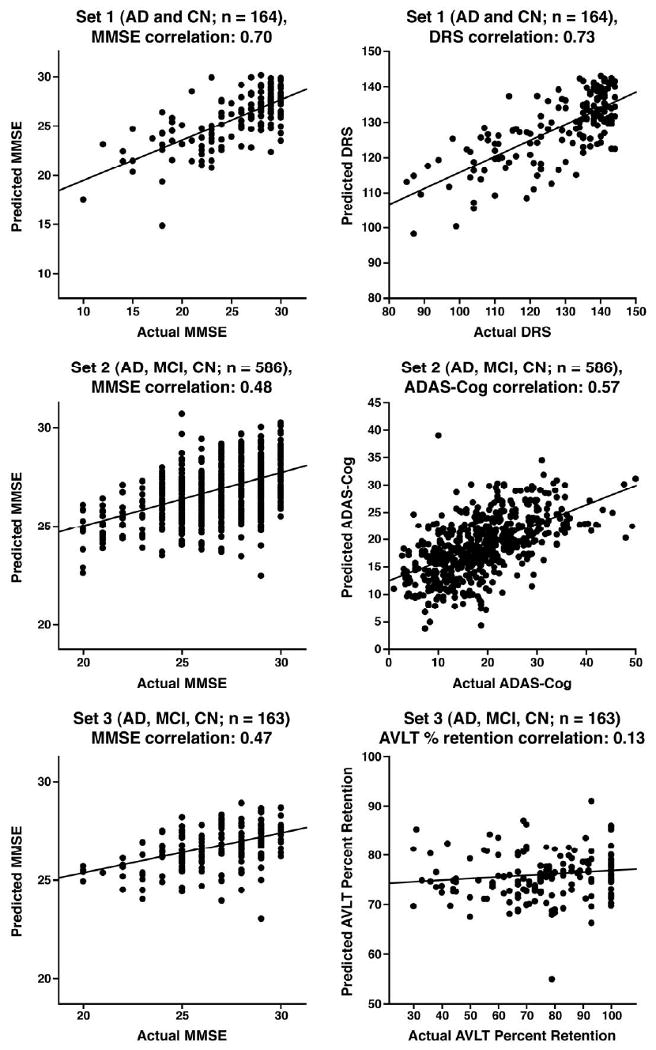
Whole brain grey matter plots of predicted versus actual scores for 4 different clinical ratings. Abbr: AD = Alzheimer's disease; MCI = Mild Cognitive Impairment; CN = Cognitively Normal; AVLT = Rey's Auditory Verbal Learning Test; DRS = Dementia Rating Scale; MMSE = Mini-Mental State Exam; ADAS-Cog = Alzheimer's Disease Assessment Scale—Cognitive Subtest.

**Fig. 4:**
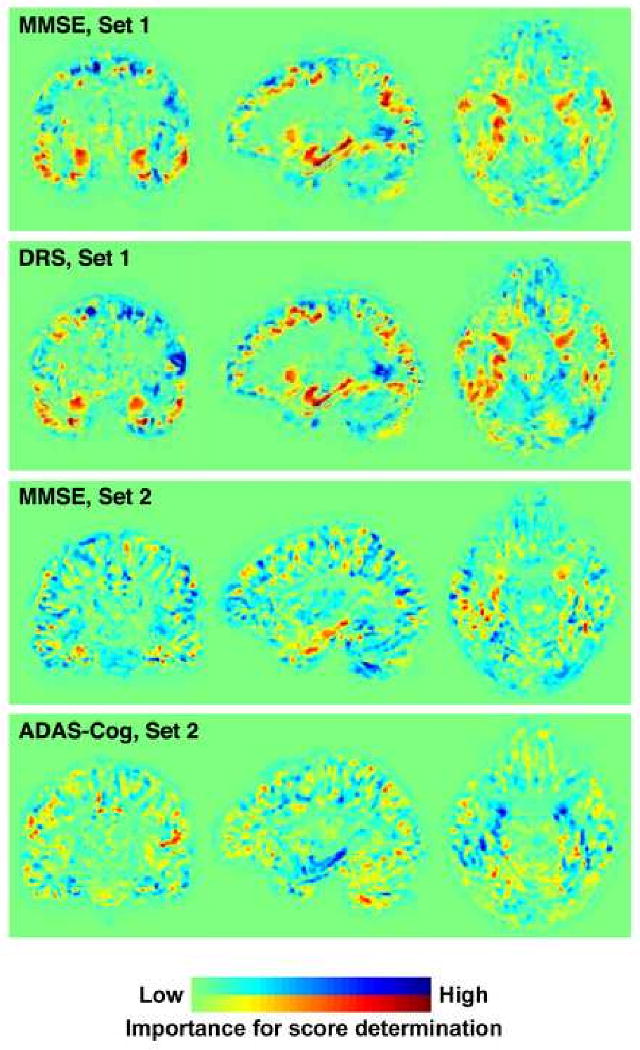
Weight maps for whole brain images, reflecting areas of the brain most vital in determining each RVR score. The red areas indicate where more grey matter adds to the accuracy of predicted test score, whereas blue areas indicate areas where more grey matter subtracts from the score. Note that dementia does not necessarily cause increased volumes of grey matter in these blue areas, but simply that data from these regions may help to adjust for anatomical variability (c.f. the contribution made by height when computing body mass index). In contrast to the MMSE and DRS, a lower score indicates better performance for the ADAS-Cog; therefore, these weight maps are mirror images. AVLT = Rey's Auditory Verbal Learning Test; DRS = Dementia Rating Scale; MMSE = Mini-Mental State Exam; ADAS-Cog = Alzheimer's Disease Assessment Scale—Cognitive Subtest.

Figure 3 contains the plots of predicted versus actual scores for the different clinical rating scales. Figure 4 depicts the weight maps for whole brain grey matter images, showing those brain areas most important for the prediction of clinical score. The RMS of the errors were as follows: MMSE: 2.19; ADAS-Cog: 7.27; AVLT: 18.97. The normalized RMS were as follows: MMSE : 0.88; ADAS-Cog: 0.87; AVLT: 1.

In addition to comparing the accuracy of predictions, we also computed the ratio of relevance vectors (number of non-zero weights divided by the total number of training samples) of each dataset and scores. The results are listed here: Set1 MMSE: 81.1%; DRS: 82.32%; AVLT: 98.17%. Set2 MMSE: 86.52%; ADAS-Cog: 99.66%. Set3 MMSE: 98.77%; ADAS-Cog : 98.77%; AVLT: 98.77%.

## Discussion

We were able to assess different clinical scores with respect to the same structural data using RVRs. Our results imply strong linear relationships between DRS, MMSE and ADAS-Cog scores and GM segments of T1 whole brain weighted images, but not with the AVLT. The normalized RMS results verify that the DRS, closely followed by MMSE in set 1, and the ADAS-Cog, closely followed by MMSE in sets 2 and 3 provided the best predictions. Whole brain images gave a better correlation with MMSE, DRS, and ADAS-Cog because they test multiple domains, unlike the AVLT. The AVLT largely tests the single domain of memory, which is associated with medial temporal lobe structures. In this case, brain regions outside this territory may have contributed relatively more noise than discriminant signal. Thus, for prediction of single domain test scores from structural images, using a well placed VOI may prove useful. Set 1 did not include an MCI group; the prediction accuracies may have been inflated by the large group of CN subjects with many scores at ceiling. The correlations were also likely stronger in Set 1 due to inclusion of more severe AD subjects, reflected by lower MMSE scores. The difference in disease severity between Sets 1 and 2 is also evident when comparing their MMSE weighted images (Figure 4). Removing the large group of CN subjects from Set 1 lowered prediction accuracies to more closely reflect those of Set 2. Conversely, when Set 1 (no MCI group and a more severe AD group) was used for training and Set 2 (large MCI group) was used for testing, the predication accuracy worsened, probably because comparable subjects were missing from the training set.

Demonstration of stability between different datasets is important for the future clinical use of machine learning methods. Training with one dataset and testing with another demonstrated stability between them when the training and testing groups were comparable, e.g., set 1 and set 2 with no MCI group, or when the training set included a wider group than the testing set, e.g. set 2 for training and set 1 for testing. Therefore, the prediction accuracy correlation is likely more trustworthy with a distributed range of scores and scans and a large number of training samples, such as in set 2 with the large group of MCI subjects in addition to AD and CN subjects. The inclusion of more severe AD subjects in the training set would likely improve the performance further due to an even wider range of both scores and structural changes.

One proposed use of prediction accuracy by RVR is to test how well a particular score correlates with structure for any disease. Future studies should evaluate RVRs of whole brain images with other instruments, such as the Short-Test of Mental Status (Kokmen et al., 1987) and the Montreal Cognitive Assessment (Nasreddine et al., 2005). Using prediction accuracy to determine which of the commonly used clinical global assessment screens are most accurately predicted from brain images of patients with MCI and early AD should prove a useful validation method of the instrument and might establish an optimal short battery of screening tests for tracking disease progression. From the individual patient perspective, this method may prove useful when clinical score data are not available. For example, predicting performance on global cognitive screening tests from an MRI scan may help to distinguish delirium from dementia in patients presenting to an emergency department with confusion and no prior records reflecting previous mental status.

There are several cautions and limitations when interpreting of our results. The results of the ratio of relevance vectors suggest that the training is not very sparse; the low sparsity suggests that information from many images contributes towards predictions, which may indicate that more scans would provide additional information. Even though statistically significant, the more modest correlations with a limited set consisting of only AD or MCI patients cautions us from drawing firm conclusions regarding the clinical significance of the procedure at this juncture. Further validation studies with differing sample sizes and ranges of disease severity will help to clarify this issue with respect to RVR. We restricted our analyses to GM segmented images. It is possible that certain clinical tests reflect white matter (WM) changes whereas others reflect GM changes (Baxter et al., 2006). However, analyses using a kernel of GM plus WM performed on the same data sets did not improve the accuracy of any predictions. Given that atrophy in GM is a more established attribute of AD, the WM images likely added more noise than useful information. Future studies that also incorporate WM hyperintensities reflecting vascular pathology, which is known to occur in parallel with GM changes, may add more useful information. Also, after eliminating subjects whose testing was outside 3 months of a scan, CN subjects were slightly younger than the AD patients in set 1. However, the contribution from age should be relatively small, and further univariate analysis in which we removed the effect of age at each voxel by treating it as a confounding variable improved rather than diminished the correlations (MMSE=0.72, DRS=0.76, and AVLT=0.63). Similarly, the slight group differences in gender distribution and education in sets 2 and 3 are unlikely to have substantially affected prediction accuracy. Furthermore, comparing prediction accuracy of one clinical test with another within the same set should not be affected by such a bias, since the prediction accuracy of each clinical score would be subject to the same inhomogeneity.

It is possible that for tests showing a good correlation with structure, the prediction accuracy (the actual score minus the predicted score) may provide useful clinical information. Since RVR gives probabilistic predictions, it is possible to measure the distance in standard deviations between predicted and actual scores. For example, in those who have learned compensatory strategies or can tolerate progressive brain pathology without manifesting cognitive symptoms, *i.e.*, have a greater cognitive reserve (Stern, 2006), the expectation would be a predicted score lower than the clinical score. Years of education—one factor thought to provide cognitive reserve—and the prediction error (actual score minus predicted score) were significantly correlated for the MMSE and ADAS-Cog in the ADNI data-set. It is interesting to note that the 3 obvious outliers in Figure 3 (Set 2) include 2 MCI subjects, each with 18 years of education: one with an ADAS-Cog score of 10, but a predicted ADAS-Cog score of 39.04 (actual MMSE of 28 and predicted MMSE of 23.47) and the other with an actual MMSE of 29 and predicted MMSE of 23.99 (actual ADAS-Cog of 23.33 and predicted ADAS-Cog of 30.14). Conversely, the third outlier was a CN subject with 12 years of education, actual MMSE of 25 and predicted MMSE of 30.68 (actual ADAS-Cog 7.33 and predicted ADAS-Cog 3.8). Visual inspection of the MRI scans for these subjects reveals more atrophy in the MCI subjects than the CN subject, indicating that the predicted score is more reflective of brain pathology than actual scores in these cases, but that factors such as educational level may have boosted performance on clinical screening tests. Furthermore, among MCI subjects with an MMSE score of 30, the predicted score was significantly lower in those who subsequently converted to AD than those who did not (*P* =0.0006). However, more studies are needed to assess this method for making predictions about cognitive reserve that include additional factors such as exercise and leisure activities; or whether fatigue or depression is a factor in subjects whose actual test scores are lower than predicted scores; as well as further evaluation and correction for the possibility that the RVR is under-estimating higher and over-estimating lower scores.

RVR offers a novel, multivariate method to test specific inter-regional dependencies between structural changes and clinical scores. As expected, and consistent with results from VBM studies, our results support the utility of the DRS, MMSE, and ADAS-Cog for screening and tracking AD. Perhaps more intriguing is RVR's ability to aid in making predictions for individual subjects. In the subset of MCI subjects from the ADNI data-set, correlation of predicted ADAS-Cog or MMSE scores with days to conversion to AD was not substantially better than the actual score. Nonetheless, it is possible that other imaging modalities might work better for this purpose and RVR may well prove useful in the prediction of imminent disease. Future studies will be directed at developing and assessing methods to combine clinical scores, with MRI, PET and CSF biomarkers for the purpose of predicting clinical outcome.

## Figures and Tables

**Fig. 1:**
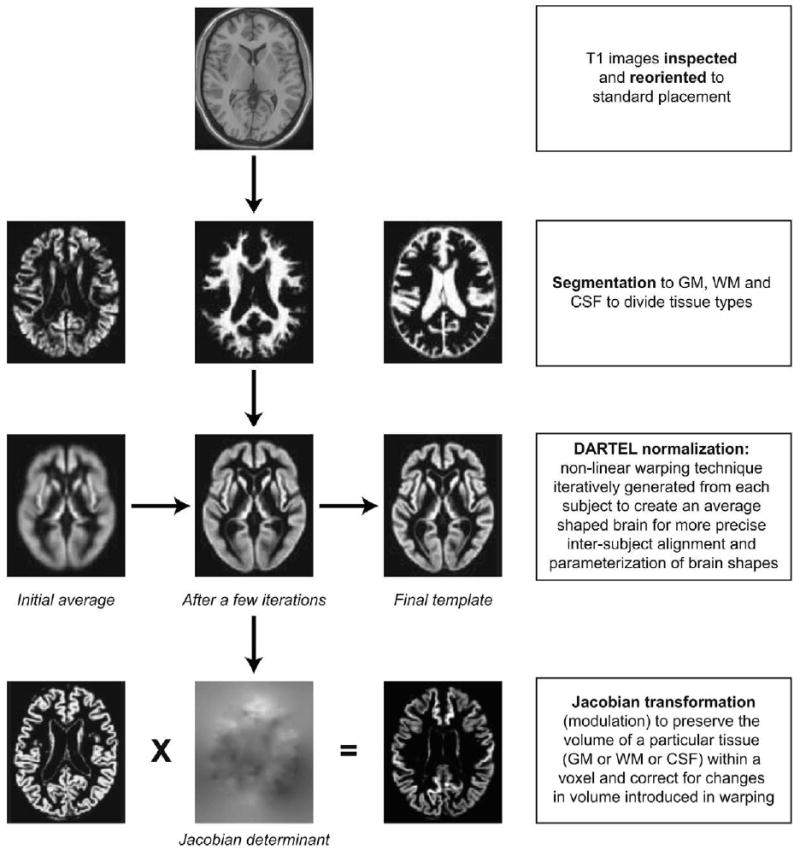
Flow diagram showing pre-processing steps. Abbr.: GM = grey matter; WM = white matter; CSF = cerebral spinal fluid; DARTEL = Diffeomorphic Anatomical Registration Through Exponentiated Lie Algebra.

**Table 1:** Demographic Information

characteristic	SET 1 Mayo Clinic, n=164	SET 2 ADNI, n=586	Set 3 ADNI, n=163
AD/MCI/CN (n)	73/0/91	113/351/122	39/92/32
Sex (F/M)	53/111	231/355	61/102
Age (mean, range)	75.9 (50-92)	75.1 (55-91)	75.3 (58-88)
Years of Education (mean, range)	14.1 (7-20)	15.5 (4-20)	15.4 (4-20)
MMSE (mean, range)	25.9 (10-30)	26.7 (20-30)	26.6 (20-30)
AVLT Percent Retention (mean, range)	8.4* (2-15)	--	75.7** (30-100)
DRS total raw score (mean, range)	128.7 (85-144)	--	--
ADAS-Cog (mean range)	--	18.90 (1-50)	19.40 (3-48)

* Percent retention, Mayo's Older Americans Normative Studies (MOANS) standard scores (Ivnik et al., 1990),

** Percent retention, raw score.

Abbr: AD = Alzheimer's disease; MCI = Mild Cognitive Impairment; CN = Cognitively normal; ADNI= Alzheimer's Disease Neuroimaging Initiative; AVLT = Rey's Auditory Verbal Learning Test; ADAS-Cog = Alzheimer's Disease Assessment Scale—Cognitive subtest; DRS = Dementia Rating Scale; MMSE = Mini-Mental State Exam
